# Evaluation of Clinical Interest of Anti-Aquaporin-4 Autoantibody Followup in Neuromyelitis Optica

**DOI:** 10.1155/2013/146219

**Published:** 2013-04-28

**Authors:** Jean-Baptiste Chanson, Melissa Alame, Nicolas Collongues, Frédéric Blanc, Marie Fleury, Gabrielle Rudolf, Jérôme de Seze, Thierry Vincent

**Affiliations:** ^1^Département de Neurologie, Hôpitaux Universitaires de Strasbourg, 1 Avenue Molière, 67091 Strasbourg, France; ^2^Laboratoire d'Imagerie et de Neurosciences Cognitives (LINC), Université de Strasbourg-CNRS, 1 rue Kirschleger, 67000 Strasbourg, France; ^3^Département d'Immunologie, Hôpital Saint-Eloi, Centre Hospitalier Universitaire de Montpellier, 80 Avenue Augustin Fliche, 34295 Montpellier, France; ^4^Institut de Génétique Humaine, CNRS UPR1142, 141, rue de la Cardonille, 34396 Montpellier, France

## Abstract

Neuromyelitis optica (NMO) is an autoimmune disease in which a specific biomarker named NMO-IgG and directed against aquaporin-4 (AQP4) has been found. A correlation between disease activity and anti-AQP4 antibody (Ab) serum concentration or complement-mediated cytotoxicity has been reported, but the usefulness of longitudinal evaluation of these parameters remains to be evaluated in actual clinical practice. Thirty serum samples from 10 NMO patients positive for NMO-IgG were collected from 2006 to 2011. Anti-AQP4 Ab serum concentration and complement-mediated cytotoxicity were measured by flow cytometry using two quantitative cell-based assays (CBA) and compared with clinical parameters. We found a strong correlation between serum anti-AQP4 Ab concentration and complement-mediated cytotoxicity (*P* < 0.0001). Nevertheless, neither relapse nor worsening of impairment level was closely associated with a significant increase in serum Ab concentration or cytotoxicity. These results suggest that complement-mediated serum cytotoxicity assessment does not provide extra insight compared to anti-AQP4 Ab serum concentration. Furthermore, none of these parameters appears closely related to disease activity and/or severity. Therefore, in clinical practice, serum anti-AQP4 reactivity seems not helpful as a predictive biomarker in the followup of NMO patients as a means of predicting the onset of a relapse and adapting the treatment accordingly.

## 1. Introduction

Neuromyelitis optica (NMO) is a severe inflammatory demyelinating disease of the central nervous system in which inflammatory lesions are usually restricted to the spinal cord and optic nerves [1, 2]. NMO is distinguished from multiple sclerosis (MS) by a more severe evolution and a predominant humoral response [3]. A highly disease-specific serum antibody (Ab), NMO immunoglobulin G (NMO-IgG), has been recently discovered [4]. The target antigen of NMO-IgG was identified as aquaporin-4 (AQP4), the main water channel protein in the CNS [5]. Owing to its very high specificity in NMO, anti-AQP4 Ab was included in the revised diagnostic criteria for NMO [6]. Anti-AQP4 Ab also proved to be helpful in predicting a more severe course and a probable conversion to NMO after a first episode of isolated optic neuritis or longitudinally extensive transverse myelitis [7–11]. Several experiments in animal models demonstrated a pathogenic role of this antibody in the disease [12–14]. Thereafter, it was questioned whether anti-AQP4 Ab serum concentration is related to disease activity in humans and, thus, whether its assessment could be helpful in predicting disease evolution and adapting the treatment.

A link between anti-AQP4 serum reactivity and various clinical parameters in NMO patients was suggested by numerous studies. Takahashi et al. found an association between high anti-AQP4 Ab serum concentration and both transverse myelitis lesion length and disease activity [15]. Accordingly, Jarius et al. [16] and Waters et al. [17] described increased anti-AQP4 serum concentration during clinical relapses and a decrease following immunosuppressive therapy. Kim et al. suggested that anti-AQP4 Ab levels correlate with disease activity but noted that rising of anti-AQP4-Ab levels did not always lead to acute exacerbation [18]. However, when testing complement-dependant cytotoxic properties of NMO patients' serum on AQP4 transfected cells, Hinson et al. observed that severe relapses were associated with higher cytotoxicity but not higher anti-AQP4 serum concentration [19]. Finally, Dujmovic et al. found that cerebrospinal fluid (CSF) but not serum anti-AQP4 Ab titers are associated with disease activity and neuroinflammation [20], whereas Jarius et al. suggested that both parameters are correlated with relapses [21].

Here, we aimed to evaluate the interest of a longitudinal assessment of anti-AQP4 Ab in clinical practice. We therefore compared the evolution of both anti-AQP4 Ab serum concentration and complement-mediated cytotoxicity with key clinical parameters such as relapse onset and disease severity.

## 2. Patients and Methods

### 2.1. Patients

Among the NMO patients followed up in the Neurology Department of the University Hospital of Strasbourg, we selected those who were positive for NMO-IgG. The disease was diagnosed according to the revised criteria of Wingerchuk et al. [6]. NMO-IgGs were detected by indirect immunofluorescence (IIF) on primate cerebellum slides with diluted sera (1/50) following the manufacturer's instructions (Instrumentation Laboratory, Lexington, MA, USA). Patients must have presented at least one relapse in the 5 last years. Ten NMO patients were included. We collected both retrospectively and prospectively serum samples taken during a relapse or a control visit. Presence of a relapse and disease severity (evaluated by the Expanded Disability Status Scale, EDSS) at the time of the blood draw were registered. Relapses were treated at least with intravenous high-dose methylprednisolone (1000 mg/day over 3–5 days) and for the most severe relapses with plasma exchanges (PE, 4-5 PE cycles). The samples were taken before corticosteroid treatment or PE. The interval between the onset of the relapse and the blood draw was from three to seven days. Presence of an immunosuppressive therapy (cyclophosphamide, mycophenolate mofetil, azathioprine, mitoxantrone, or rituximab) was also registered. Written informed consent was obtained from all patients. Study protocol was declared to the CNIL (Commission Nationale Informatique et Libertés), and the study was performed in accordance with the ethical standards laid down in the 1964 Declaration of Helsinki.

### 2.2. Measurement of Anti-AQP4 Ab Serum Concentration

Sera were collected between 2006 and 2011 and stored at −80°C. They were sent to the Department of Immunology of the University Hospital of Montpellier (France). Anti-AQP4 Ab serum concentration was measured without knowledge of clinical information using a new quantitative cell-based assay (CBA) recently validated [22]. Briefly, HEK-293 T human cell line was transduced using HIV-based vectors expressing either the human *AQP4* and enhanced green fluorescent protein (*EGFP*) genes (293-AQP4/EGFP cells) or the *EGFP* gene alone (293-EGFP cells). Cells were incubated with diluted human sera (1/200) in FACS buffer (PBS containing 1% fetal calf serum and 0.1% sodium azide), washed, and incubated with allophycocyanin- (APC-)conjugated goat anti-human IgG Ab (Jackson ImmunoResearch Laboratories, West Grove, USA). After washes, cells were analyzed on a FACS Calibur (BD Biosciences), and APC-mean fluorescence intensity (MFI) was measured on EGFP positive cells. A serum was considered anti-AQP4 positive if it bound 293-AQP4/EGFP but not 293-EGFP cells. To determine the anti-AQP4 concentration (arbitrary units; AU/mL), the APC-MFI of 293-AQP4/EGFP cells incubated with 1/200 diluted patient sera and corrected for the background binding to the 293-EGFP control cells was plotted on a calibration curve obtained with a pool of NMO-IgG positive sera diluted from 1/200 (1000 AU/mL) to 1/51200 (3.9 AU/mL). All sera from the same patient were analyzed simultaneously.

### 2.3. Measurement of Complement-Mediated Serum Cytotoxicity

The protocol was inspired from Hinson et al. [19]. 293-AQP4/EGFP and 293-EGFP cells were incubated with patients' sera (20% serum in FACS buffer for 30 minutes at +4°C). After washes, cells were incubated with active complement (40% complement for 45 minutes at 37°C; Low-Tox-H rabbit complement; Cedarlane Laboratories Ltd, Burlington, NC, USA) and complement-dependent cytotoxicity (CDC) was measured by flow cytometric analysis of the percentage of EGFP positive cells permeable to the viability dye propidium iodide (PI). A pool of normal sera from healthy subjects was included in each experiment as a negative control. Serum cytotoxicity was defined (AU/mL) by the difference between the percentage of PI positive cells induced in 293-AQP4/EGFP cells versus 293-EGFP cells corrected by the background of mortality obtained with the pool of normal sera. All sera from the same patient were analyzed simultaneously.

### 2.4. Statistical Analysis

The Pearson correlation coefficient was used to search for a correlation between anti-AQP4 Ab serum concentration and cytotoxicity. The nonparametric Mann-Whitney *U* test was used to determine whether anti-AQP4 serum concentration or cytotoxicity was related to the presence of a relapse or disease severity. We compared both biological parameters in samples taken in relapsing phase (<1 month after a relapse) versus remitting phase and in the 5 more disabled patients (i.e., with the highest mean EDSS scores) versus the other ones. A Bonferroni correction for multiple comparisons was applied and the statistical significance was defined as *P* value of *P* < 0.01.

## 3. Results

### 3.1. Patients

Thirty sera were collected from 10 NMO-IgG positive patients (mean: 3 per patient, range 2–5) between 2006 and 2011. Patients' characteristics are summarized in Table 1. All were women. Ten samples (33%) were taken during a relapse. The mean interval between two samplings was 10.7 ± 8 months (mean ± standard deviation).

### 3.2. Evaluation of Anti-AQP4 Ab Serum Concentration and Complement-Dependent Cytotoxicity

**Figure 1:**
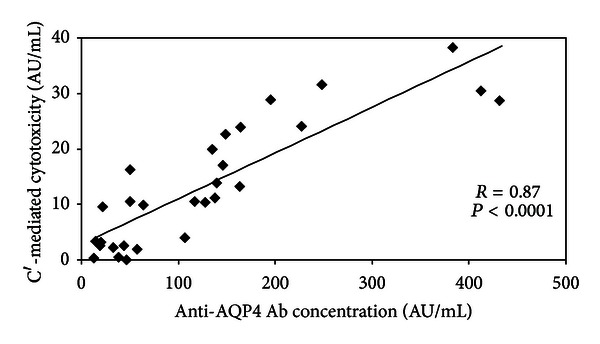
Correlation between serum anti-AQP4 Ab concentration and serum complement- (*C*′-)mediated cytotoxicity. Anti-AQP4 Ab serum concentration and complement- (*C*′-)mediated serum cytotoxicity were measured in 30 sera from 10 NMO patients with a cell-based assay using AQP4 expressing human 293 T cells and flow cytometry. Results are expressed in arbitrary units (AU/mL). The Pearson correlation coefficient (*r*) was 0.87 (*P* < 0.0001).

As some studies described discrepancies between anti-AQP4 Ab serum concentration and complement-dependent cytotoxicity, we measured and compared these parameters in different NMO patients' sera. Mean anti-AQP4 Ab serum concentration was 158 ± 136 AU/mL (range 132–>1000) and mean cytotoxicity was 14.1 ± 11 AU/mL (range 0–38.8). We observed a high intra- and interindividual variability especially in anti-AQP4 Ab serum concentration. However, as shown in Figure 1, serum anti-AQP4 Ab concentration and complement-dependent cytotoxicity were highly correlated (*r* = 0.87, *P* < 0.0001).

### 3.3. Relationships between Biological and Clinical Parameters

**Figure 2:**
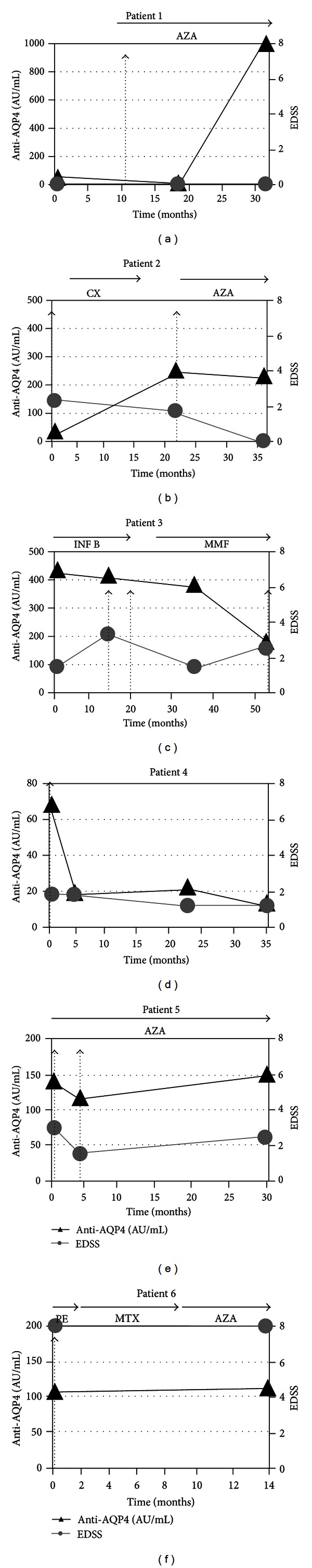
Detailed evolution of anti-AQP4 serum reactivity and comparison with disease course in patients 1 to 6. Serum anti-AQP4 Ab concentration (black triangle) was measured with a cell-based assay using AQP4 expressing human 293 T cells and flow cytometry as described in Section 2. EDSS score indicates the level of impairment (grey circle); vertical dashed arrows represent relapses (all relapses were treated with corticosteroids); horizontal arrows indicate a treatment (AZA: azathioprine; CX: monthly administration of IV corticoids; INF B: interferon beta; MMF: mycophenolate mofetil; PE: plasma exchange; MTX: mitoxantrone).

**Figure 3:**
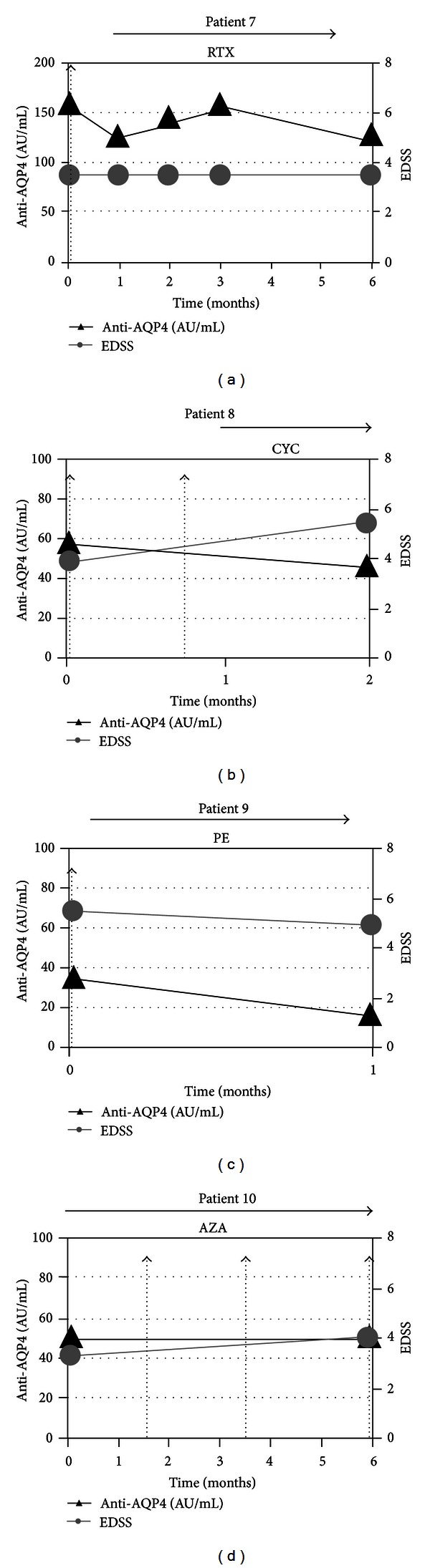
Detailed evolution of anti-AQP4 serum reactivity and comparison with disease course in patients 7 to 10. Serum anti-AQP4 Ab concentration (black triangle) was measured with a cell-based assay using AQP4 expressing human 293 T cells and flow cytometry as described in Section 2. EDSS score indicates the level of impairment (grey circle); vertical dashed arrows represent relapses (all relapses were treated with corticosteroids); horizontal arrows indicate a treatment (RTX: rituximab (1 perfusion weekly of 375 mg/m^2^ during 4 weeks); CYC: cyclophosphamide; PE: plasma exchange; AZA: azathioprine).

Detailed anti-AQP4 reactivity course was compared to relapse onset, EDSS changes, and treatment for each patient as shown in Figures 2 and 3. As summarized in Table 2, we did not observe any significant correlation between biological parameters and disease activity. Indeed, increase in anti-AQP4 Ab concentration may occur with no clinical change (e.g., patient 1), and conversely disease worsening was often not accompanied by an increase in anti-AQP4 Ab levels (patients 2 and 3). A significant effect of immunosuppressant was noted on anti-AQP4 Abs concentration. Indeed, mycophenolate mofetil initiation was concomitant with a marked decreased anti-AQP4 reactivity in patient 3. A less significant decrease was observed under azathioprine in patient 2 and under cyclophosphamide in patient 6. Plasma exchanges resulted in a rapid anti-AQP4 reactivity decline (patient 9). However, a similar effect was not observed under azathioprine in patients 1 and 6. Rituximab initiation in patient 7 resulted in an early increase (with no clinical event) followed by a delayed decrease (at 6 months) in anti-AQP4 reactivity.

As shown in Table 2, statistical analysis found no relationship between anti-AQP4 serum concentration or complement-mediated cytotoxicity and the presence of a relapse or disease severity (EDSS score).

## 4. Discussion

The followup of our patients suggests that anti-AQP4 Ab serum concentration and complement-mediated cytotoxicity are strongly correlated, but that their monitoring does not allow an accurate and reliable prediction of disease evolution.

The only study that longitudinally assessed serum complement-mediated cytotoxicity in NMO patients reported that this parameter was better correlated with disease activity than anti-AQP4 Ab serum concentration [19]. These findings were not in accordance with the marked link previously demonstrated in vitro between anti-AQP4 Ab concentration and complement-dependent cytotoxicity [23, 24]. In our patients, despite high inter- and intraindividual variability, we found a marked correlation between anti-AQP4 Ab serum concentration and complement-mediated serum cytotoxicity (*P* < 0.0001). This result suggests that measurement of serum cytotoxicity does not give an extra insight compared to anti-AQP4 Ab concentration assessment. Accordingly, when compared with disease course or severity, anti-AQP4 Ab serum concentration and complement-mediated cytotoxicity gave very similar and nonsignificant results.

NMO is characterized by severe relapses often resulting in sustained functional impairment and it would be of particular interest to predict a relapse onset for an earlier adaptation of the treatment [25]. Unfortunately, in the followup of our patients, relapses were rarely associated with relevant changes in anti-AQP4 reactivity. This is well illustrated by patients 2 and 3 in which anti-AQP4 reactivity was lower during a relapse than a remitting phase. Conversely, we observed in other cases that marked changes in anti-AQP4 reactivity were not accompanied by any clinical event (e.g., patient 1). A potential confounding factor was the presence of immunosuppressive therapies which are often prescribed in NMO [15, 16]. We frequently observed a decrease in anti-AQP4 Ab concentration after the beginning of an immunosuppressive treatment. However, this effect was not systematic as was shown in patients 1 and 6. Transient increase in anti-AQP4 reactivity observed after Rituximab initiation in patient 7 is consistent with those reported in two recent papers [26, 27]. Interestingly, these studies did not observe any correlation between the increase in anti-AQP4 Ab serum concentration and the worsening of the disease.

Our results do not preclude a pathogenic role of serum anti-AQP4 Ab in NMO but underline the requirement of additional factors to allow peripheral Abs to cross the blood-brain barrier (BBB) and gain their targets in the CNS. Indeed, the worsening of experimental autoimmune encephalitis after intraperitoneal injection of anti-AQP4 Abs in animal was dependant on a previous BBB breakdown and the presence of inflammatory cytokines [12]. Likewise, intrathecal or intracerebral injection of NMO-IgG in the CNS induces NMO-like lesions, whereas peripheral injection does not cause CNS damage [14]. Indeed, although tolerance breakdown is thought to occur in the periphery, pathogenic processes occur in the CNS. Hence, as suggested by recent studies, it might be more relevant to use CSF rather than serum anti-AQP4 titer as a biomarker for the followup of NMO patients [20, 28].

Our study presented some limitations that should be pointed out. The relative small number of patients tested may have limited the detection of significant correlations between immunological and clinical parameters. This study cannot address in detail the question whether relapses are preceded by an increase in AQP4 serum levels, since no paired samples taken shortly before and during relapse onset were available for comparison from most patients. Due to the retrospective design, time intervals between the samplings and treatments of patients were variable. The statistical results must be therefore interpreted with caution. However, this heterogeneity reflects the clinical practice in which patients are not monitored with short and regular intervals and receive various drugs. At an individual level, we noted that anti-AQP4 Ab concentration is highly variable and is related to many factors including the administration of immunosuppressants. However, it did not appear closely correlated with disease activity.

## 5. Conclusions

Altogether, our results showed that anti-AQP4 serum concentration in NMO patients is representative of serum complement-mediated cytotoxicity. The lack of strong correlation between the anti-AQP4 serum reactivity and disease course in our study suggests that anti-AQP4 Ab monitoring could not be used to reliably predict relapses and adapt treatment. Therefore, the longitudinal evaluation of serum anti-AQP4 reactivity does not appear helpful for an accurate followup of NMO patients in clinical practice.

## Figures and Tables

**Table 1:** Clinical features of NMO patients.

Patient number	Age (yrs)	Disease duration (yrs)	Follow-up duration (mo)	EDSS	Immunosuppressive treatment
1	54	4	31	0	AZT
2	19	2	37	2.5	AZT
3	38	6	53	1.5	MMF
4	61	1	35	2	None
5	19	1	30	3	AZT
6	63	12	14	8	MTX then AZT
7	27	3	6	3.5	RTX
8	46	0.1	2.5	4	CYC
9	62	1.5	0.5	5.5	PE
10	54	1	6	3.5	AZT

Mean ± SD	44.3 ± 17.5	3.2 ± 3.8	26.5 ± 16	3.4 ± 3	

yrs: years; mo: months; disease duration: duration from the first symptom of the disease to the first blood draw; follow-up duration: duration from the first to the last blood draw; EDSS: EDSS score evaluating disease severity; AZT: azathioprine; MMF: mycophenolate mofetil; RTX: rituximab; MTX: mitoxantrone; CYC: cyclophosphamide; PE: plasma exchanges.

**Table 2:** Relationship between anti-AQP4 Ab serum concentrations, complement-mediated cytotoxicity, and clinical parameters.

	Anti-AQP4 concentration (AU/mL)	*P *	Serum cytotoxicity (AU/mL)	*P *
Patients during a relapse	124 ± 127	*P* = 0.71	13.5 ± 12	*P* = 0.89
Patients during a remitting phase	176 ± 224		14.2 ± 11	
Patients with the more severe impairment	94 ± 52	*P* = 0.38	10.2 ± 7.7	*P* = 0.21
Patients with the milder impairment	208 ± 248		17 ± 13	

Anti-AQP4 Ab serum concentration and complement-mediated serum cytotoxicity were measured in 30 sera from 10 NMO patients with a cell-based assay as described in methods. We compared both biological parameters in samples taken in relapsing phase (<1 month after a relapse) versus remitting phase and in the 5 more disabled patients (i.e., with the highest EDSS scores) versus the other ones. *P*: alpha risk.
